# Integrative and complementary practices to control nausea and vomiting in pregnant women: a systematic review^*^

**DOI:** 10.1590/1980-220X-REEUSP-2021-0515en

**Published:** 2022-10-21

**Authors:** Melissa Santos Nassif, Isabelle Cristinne Pinto Costa, Patricia Mônica Ribeiro, Caroline de Castro Moura, Paloma Elisama de Oliveira

**Affiliations:** 1 Universidade Federal de Alfenas, Escola de Enfermagem, Alfenas, MG, Brazil.; 2 Universidade Federal de Viçosa, Departamento de Medicina e Enfermagem, Viçosa, MG, Brazil.

**Keywords:** Pregnancy, Nausea, Vomiting, Complementary Therapies, Systematic Review, Embarazo; Náusea, Vómitos, Terapias Complementarias, Revisión Sistemática, Gravidez, Náusea, Vômito, Terapias Complementares, Revisão Sistemática

## Abstract

**Objective:**

to synthesize the evidence available in the literature on the effects of integrative and complementary practices in nausea and vomiting treatment in pregnant women.

**Method:**

a systematic review, reported according to PRISMA and registered in PROSPERO. The search for studies was carried out in 11 databases. To assess risk of bias in randomized clinical trials, the Cochrane Collaboration Risk of Bias Tool (RoB 2) was used.

**Results:**

the final sample consisted of 31 articles, divided into three categories: aromatherapy, phytotherapy and acupuncture. It was observed that aromatherapy with lemon essential oil, ginger capsules, pericardial 6 point acupressure were the interventions that proved to be effective. Less than half of studies reported adverse effects, with mild and transient symptoms predominating. Most articles were classified as “some concern” in risk of bias assessment.

**Conclusion:**

the three most effective interventions to control gestational nausea and vomiting were aromatherapy, herbal medicine and acupuncture, with significant results in the assessment of individual studies.

## RESUMO

**Objetivo:**

sintetizar as evidências disponíveis na literatura sobre os efeitos das práticas integrativas e complementares no tratamento de náusea e vômito em gestantes.

**Método:**

revisão sistemática, relatada conforme o PRISMA e registrada no PROSPERO. A busca pelos estudos foi realizada em 11 bases/bancos de dados. Para avaliação do risco de viés dos ensaios clínicos randomizados, utilizou-se a ferramenta *Cochrane Collaboration Risk of Bias Tool* (RoB 2).

**Resultados:**

a amostra final foi composta por 31 artigos, divididos em três categorias: aromaterapia, fitoterapia e acupuntura. Observou-se que a aromaterapia com óleo essencial de limão, cápsulas de gengibre, acupressão no ponto pericárdio 6 foram as intervenções que se provaram eficazes. Menos da metade dos estudos relatou efeitos adversos, sendo que predominaram sintomas de leve intensidade e transitórios. A maioria dos artigos foi classificada como “alguma preocupação” na avaliação do risco de viés.

**Conclusão:**

as três intervenções mais eficazes para controle de náusea e vômito gestacional foram aromaterapia, fitoterapia e acupuntura, com resultados significativos na avaliação dos estudos individuais.

## RESUMEN

**Objetivo:**

sintetizar las evidencias disponibles en la literatura sobre los efectos de las prácticas integradoras y complementarias en el tratamiento de náuseas y vómitos en gestantes.

**Método:**

revisión sistemática, reportada según PRISMA y registrada en PROSPERO. La búsqueda de estudios se realizó en 11 bases/bases de datos. Para evaluar el riesgo de sesgo en los ensayos clínicos aleatorios, se utilizó la herramienta Cochrane Collaboration Risk of Bias Tool (RoB 2).

**Resultados:**

la muestra final estuvo compuesta por 31 artículos, divididos en tres categorías: aromaterapia, fitoterapia y acupuntura. Se observó que la aromaterapia con aceite esencial de limón, las cápsulas de jengibre, la acupresión en el punto 6 del pericardio fueron las intervenciones que demostraron ser efectivas. Menos de la mitad de los estudios reportaron efectos adversos, predominando los síntomas leves y transitorios. La mayoría de los artículos se clasificaron como “cierta preocupación” en la evaluación del riesgo de sesgo.

**Conclusión:**

las tres intervenciones más efectivas para controlar las náuseas y los vómitos gestacionales fueron la aromaterapia, la fitoterapia y la acupuntura, con resultados significativos en la evaluación de los estudios individuales.

## INTRODUCTION

The pregnancy process is accompanied by several physiological changes that allow fetal development and, consequently, cause signs and symptoms for women^(1)^. Among the changes, nausea and vomiting stand out, which have multifactorial pathogenesis and usually occur between the sixth and twelfth week of pregnancy^(2)^, and affect about 50% to 80% of pregnant women^(1)^.

These changes are often associated with negative effects on the mother, affecting quality of life and marital, maternal and social relationships, which may be decisive for postpartum depression, regardless of the pre–pregnancy maternal emotional status^(3)^. There may also be feelings of inadequacy, anxiety and stress, a greater probability of having severe pain in the pelvic girdle, hypertension, preeclampsia and proteinuria^(4)^.

Currently, conventional treatment is predominantly based on dietary changes and antinauseant, antiemetic and antihistamine drugs^(2)^. The gold standard treatment is of pyridoxine (vitamin B6) use alone or associated with doxylamine^(5)^. However, pregnant women may be reluctant to use these drugs, since they were related to some side effects^(6)^. Added to this is the fact that most pregnant women claim to be afraid of the possible teratogenic effects due to the repetitive use of drugs during pregnancy^(7)^.

An approach that could contribute to reducing the consumption of medicines in this population and, consequently, the side effects, refers to Integrative and Complementary Practices in Health (ICPH), which have the potential to reduce medical expenses, cost savings, medicines and health services^(2,7)^. These are defined as “Health practices based on the model of humanized care and focused on the comprehensiveness of individuals, which seek to stimulate the natural mechanisms of disease prevention, health promotion and recovery through effective and safe technologies”^(8)^. They focus on the biopsychosocial care model, which encompasses factors sometimes neglected in biomedical practice, providing holistic and comprehensive care^(2,7)^.

In 2006, with the objective of guaranteeing comprehensive health care, the Brazilian National Policy on Integrative and Complementary Practices (PNPIC – *Política Nacional de Práticas Integrativas e Complementares*) in the Unified Health System (*Sistema* Único *de Saúde*) was approved. Currently, PNPIC offers 29 ICPH, free of charge, in 54% of Brazilian municipalities, mainly in Primary Health Care (78%), which made Brazil a reference in the area of ICPH at this level of care^(9)^.

ICPH use has become increasingly popular in Western society^(10)^. Regarding the universe of obstetrics, the same scenario is observed, according to a research that sought to map the prevalence of this use, identifying that 45%^(7)^ of pregnant women used some type of ICPH.

Considering that a high number of pregnant women are affected by nausea and vomiting and ICPH have the potential to control nausea and vomiting in this public, the main objective of this study was to synthesize the evidence available in the literature on the effects of ICPH in nausea and vomiting treatment in pregnant women.

## METHOD

This is a systematic literature review, reported according to the Preferred Reporting Items for Systematic Reviews and Meta–Analyses (PRISMA)^(11)^, registered in the International Prospective Register of Systematic Reviews (PROSPERO) CRD42020221570.

### Research Question Development

The PICO strategy (P (population) – pregnant women; I (intervention) – ICPH; C (comparison) – placebo, routine treatment and pharmacological intervention; O (outcomes) – control of nausea and vomiting and adverse effects from treatment)^(12)^ guided the research question: in pregnant women, do ICPH have an effect on the control of nausea and vomiting compared to placebo, routine treatment or pharmacological intervention?

### Eligibility Criteria

We included Randomized Clinical Trials (RCTs) with healthy pregnant women, at any gestational age (GA), who had nausea and vomiting. Parturient and puerperal women were excluded. Studies involving symptoms characterized as hyperemesis gravidarum were not included, as this represents the pathological form of nausea and vomiting of pregnancy^(2)^.

Studies were analyzed that sought to control nausea and vomiting in pregnant women, through ICPH, and were previously proven effective for pregnant women, arising from current systematic reviews, even for other study purposes, namely: aromatherapy with lemon had an impact on significantly in the control of nausea and vomiting in pregnancy^(13)^; hypnotherapy has proven to be effective in improving pregnant women’s perspectives and emotional experiences regarding childbirth^(14)^; homeopathy and herbal medicine were tested for safety of use during pregnancy^(15)^; acupuncture has been shown to be effective in controlling insomnia in pregnancy^(16)^; ear acupuncture (AA) was effective for pregnancy–related low back pain compared to placebo^(17)^; music therapy was able to significantly reduce gestational anxiety^(18)^; and yoga was efficient to reduce depressive and anxious symptoms in pregnant women^(19)^. It should be noted that the intervention was considered if used alone or as an adjunct to other methods.

As a control, we considered any placebo method (specific for each ICPH), vitamin B6 alone or associated with doxylamine, considered the gold standard in managing nausea and vomiting in pregnancy^(5)^, other pharmacological interventions and other non–pharmacological interventions. The primary results assessed were nausea and vomiting in pregnant women at any GA. The secondary result was possible adverse events arising from the use of these therapies.

It is emphasized that, currently, there is no Core Outcome Set (COS) that standardizes the forms of assessment and, in the absence of COS, it was decided to assess all the instruments proposed by included studies.

### Information Sources

The search for studies, comprised from December 2020 to January 2021, was made from of the databases as follows: Medical Literature Analysis and Retrieval System Online (MEDLINE) via PubMed, EMBASE (via Embase.com), Cumulative Index to Nursing and Allied Health Literature (CINAHL), Cochrane Central Register of Controlled Trials (CENTRAL), Web of Science, Scopus, World Health Organization International Clinical Trials Registry Platform (ICTRP), Latin American Literature in Health Sciences (LILACS), National Medical Sciences Information Center of Cuba (CUMED), Spanish Bibliographic Index of Health Sciences (IBECS), Brazilian Registry of Clinical Trials (ReBEC) and China National Knowledge Infrastructure (CNKI). There was no restriction regarding year of publication or language.

### Search Strategy

**Chart 1:**
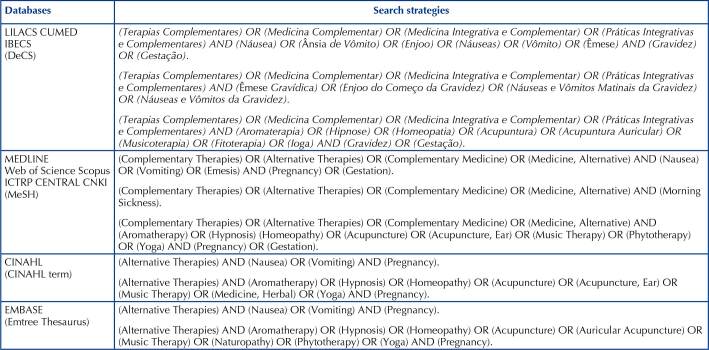
Search strategies used according to selected database – Alfenas, MG, Brazil, 2021.

Based on the research question and with the help of a librarian, terms were selected in the Descriptors in Health Sciences (DeCS) and Medical Subject Headings (MeSH), which contained the appropriate descriptors for searching in the databases. To combine these, the Boolean operators OR and AND were used, as shown in Chart 1.

### Article Selection and Assessment

Article search was performed in the aforementioned databases and then uploaded to EndNote®, whose duplicate studies were identified and removed. Then, this database was exported to Rayyan®, through which the study selection process took place. This tool allows the selection of articles by independent reviewers, with the option of blinding between them.

Articles were selected by two reviewers, independently, and, in the end, the disagreements were resolved by a third researcher, after mutual discussion. The article selection process was carried out in three stages. The first involved reading the title to find the keywords that signaled the use of some type of ICPH in pregnant women. The second phase involved reading the abstract in order to filter studies that addressed nausea and vomiting control. Finally, in the third stage, pre–selected articles were read in full and checked for pre–defined eligibility criteria. Study search, screening and selection was presented by the PRISMA flowchart^(11)^.

### Data Analysis and Treatment

After the study selection process, data collection was performed using a data extraction form, adapted for this review, based on the model proposed by the Cochrane Handbook for Systematic Reviews of Interventions^(12)^. This was based on study identification, design and methods. Information about participants, the intervention, outcome measures used and the results obtained by the studies were also collected^(20)^.

Training was carried out on how to fill out the form, reviewed and discussed by the authors, based on the extraction of data from a study, in order to unify understanding and minimize inconsistencies. Two independent researchers performed the data extraction, and a third researcher was consulted in case of disagreement. Another reviewer was in charge of crossing this information.

When data display was incomplete, the corresponding authors of the studies were contacted by email (maximum of three attempts) to complement the information.

Eligible articles were assessed regarding report quality, using the Consolidated Standards of Reporting Trials (CONSORT) checklist, which aims to assist in RCT reporting, enabling greater transparency and reproducibility of research^(21)^. Regarding risk of bias, through the Cochrane Risk of Bias Tool ROB2)^(22)^, instrument that assesses six different domains for each study in relation to risk of bias, classifying risks as “high”, “some concerns” and “low”, enabling the visualization of the reliability of the results of the analyzed studies. This assessment was performed by two independent authors, and a third was consulted to resolve possible discrepancies.

## RESULTS

**Figure 1:**
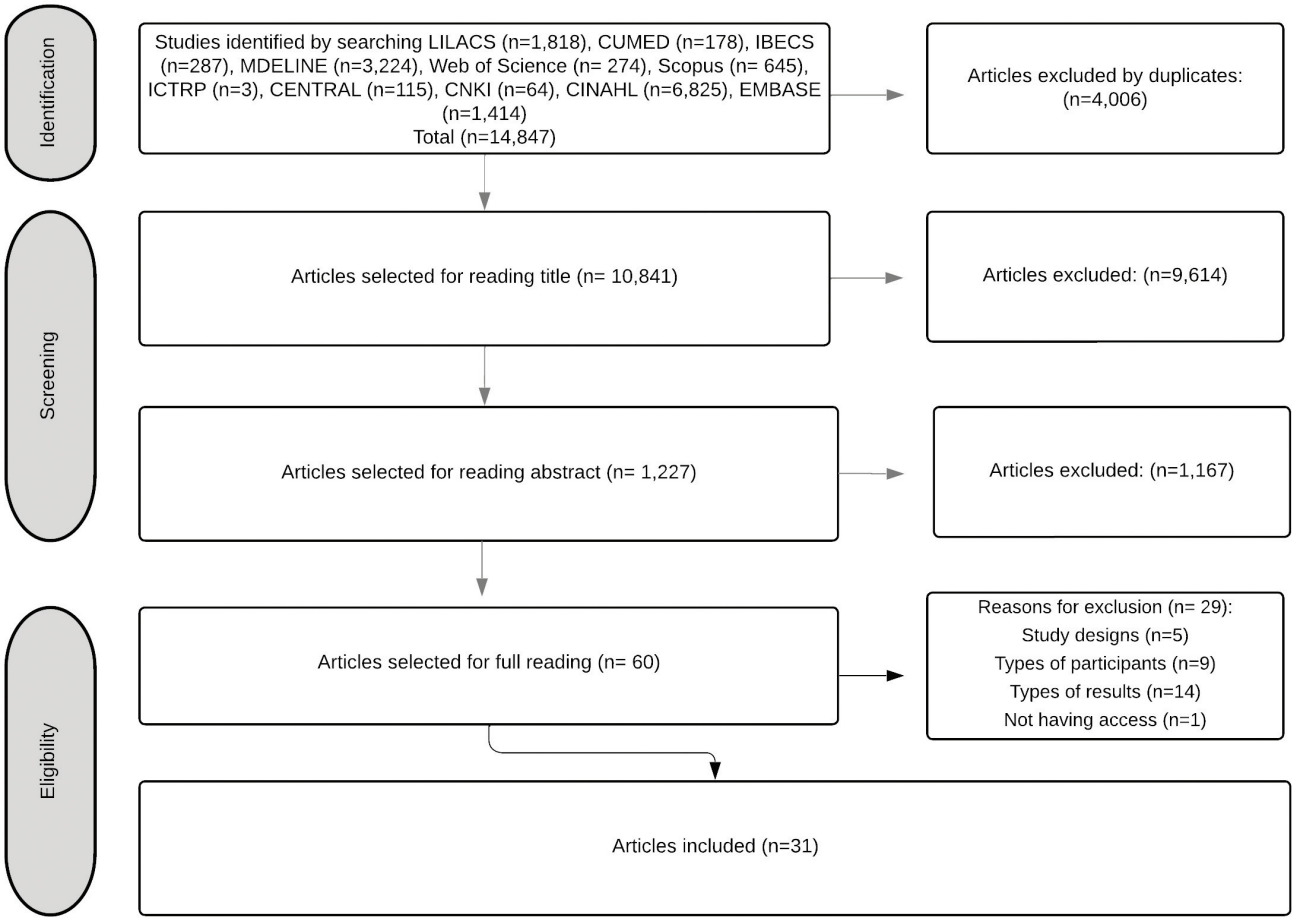
PRISMA flowchart.

The quantitative results of the study selection stages are in the following flowchart (Figure 1), according to PRISMA^(11)^.

When assessing the final sample of articles included, it is observed that the five countries that most published on the subject were Iran (n = 9)^(13,23–30)^, Thailand (n = 4)^(6,15,31,32)^, United States of America (n = 4)^(33–36)^, Australia (n = 4)^(37–40)^ and Canada (n = 4)^(41–44)^. An article was not published in English^(25)^, and the studies were published between 1988 and 2020.

Participants’ age ranged from 16 to 44 years, and GA varied from four to 23 weeks, with most studies considering a minimum GA of six weeks (n = 11)^(23–25,27–29,32,35,36,45,46)^ and a maximum of 12 (n = 8)^(30,32,35,41–43,47,48)^. The studies analyzed used random allocation. However, most studies (n = 21)^(13,15,23,25–30,33,34,39–43,45,48–51)^ partially reported the mode of execution of allocation (e.g., software), and did not describe how it was concealed (n = 18)^(13,24–29,32,33,35,38,39,41,45,47–49,51)^ (e.g., opaque envelope). It is also added that six studies detailed some form of masking^(13,23–25,33,41)^. Most clinical trials (n = 19) reported some form of blinding^(6,15,23,25,27–30,34–39,41,43,46,47,49)^, either from intervention applicators or evaluators.

Twenty different instruments used to measure outcomes were identified. Even in the face of such variability of instruments, some were used more frequently: Likert Scale (n = 4)^(15,26,41,46)^; Pregnancy–Unique Quantification of Emesis and Nausea (PUQE) (n = 5)^(23–25,27,45)^; Visual Analogue Scale (VAS) (n = 9)^(13,26,30,31,34,40,46,47,50)^; Rhodes Index of Nausea, Vomiting and Retching (n = 12)^(6,28,32,35,37–39,43,44,48,50,51)^; and diary variations (n = 12)^(26,31,33,35,40–42,44,47,49–51)^.

**Chart 2:**
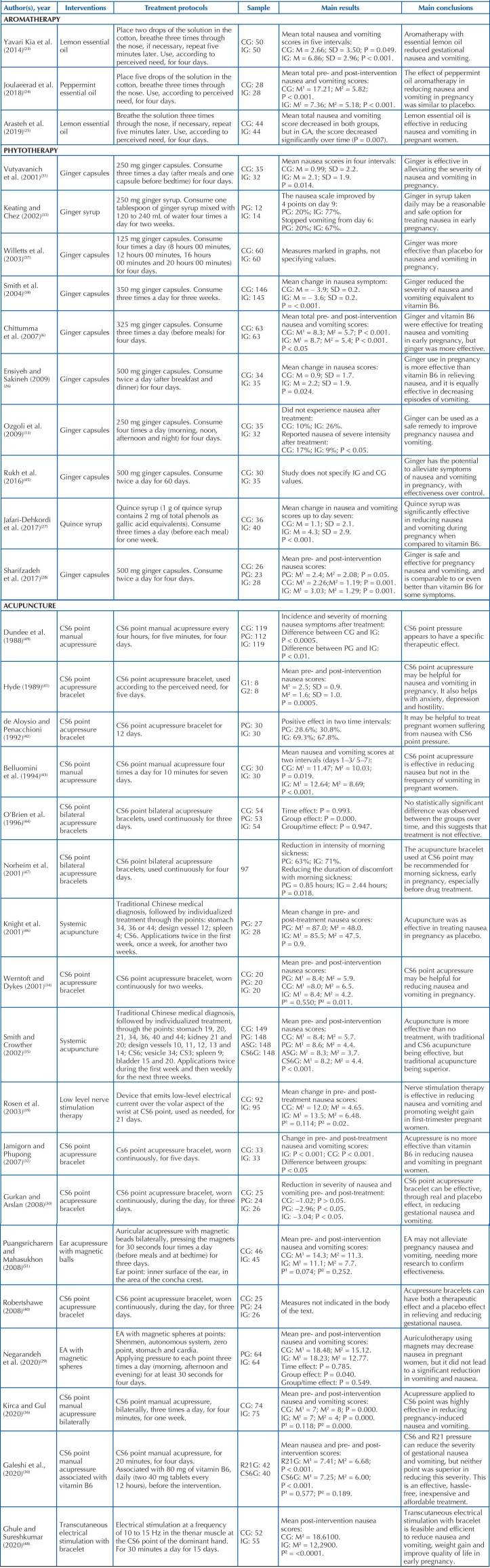
Synthesis of the main findings of studies included in the final sample (n = 31). Alfenas (MG), Brazil, 2021.

Out of the eight ICPH surveyed, aromatherapy, phytotherapy, acupuncture and EA presented results. For data presentation, the studies were grouped according to the ICPH used: aromatherapy, phytotherapy and acupuncture (Chart 2). Regarding aromatherapy, in 67% (n = 2)^(23,25)^ of studies, this group obtained superior results when compared to the control group, and 33% (n = 1)^(24)^ did not present differences between the groups. Positive results were achieved by using lemon essential oil. Regarding adverse effects, 67% (n = 2)^(23,25)^ of studies reported no adverse effects, and in 33% (n = 1)^(24)^, headache, vertigo and dyspnea were observed. These were mild in intensity, transient and did not affect the study continuity.

CS – circulation sex; SD – standard deviation; g – gram; CG – control group; IG – intervention group; PG – placebo group; Hz – hertz; M – mean; M1 – pre-intervention mean; M2 – post-intervention mean; mg – milligram; P – probability; K – kidney; EA – ear acupuncture.

In the phytotherapeutic interventions, it was possible to observe that 90% (n = 9)^(6,13,26–28,31,37,38,45)^ of studies presented results superior to the control, with 88.8% of these (n = 8)^(6,13,26,28,31,37,38,45)^ using some form of ginger, and the others, 11.1% (n = 1)^(27)^, quince. Regarding adverse effects, 10% (n = 1)^(28)^ did not mention this topic in the study; 30% (n = 3)^(13,27,33)^ did not identify adverse effects attributed to the intervention; and 60% (n = 6)^(6,26,31,37,38,45)^ reported the occurrence of headache^(6,31)^, abdominal discomfort^(31,45)^, heartburn^(6,31)^, diarrhea^(31)^, miscarriage^(26,37)^, treatment intolerance^(37)^, worsening of clinical picture^(37)^, allergic reaction^(37)^, problems swallowing^(38)^, sedation^(6)^, arrhythmia^(6)^, and xerostomia^(45)^.Most studies^(6,26,31,38,45)^ explained that participants did not discontinue participation due to these adverse events, and there was no statistical difference between groups regarding these variables.

Regarding acupuncture, 77.7% of studies (n = 14)^(29,30,34–36,39–43,47–50)^ obtained a superior result to the control group. These results were achieved by pericardium or circulation sex (CS) 6 (Nei–Guan) point (n = 11)^(30,34,36,39–43,47,49,50)^ and kidney (R) 21 (n = 1)^(30)^ acupressure, in addition to EA (n = 1)^(29)^, systemic acupuncture (n = 1)^(39)^ and nerve stimulation (n = 2)^(35,48)^. It is noteworthy that some studies assessed more than one type of intervention. It is worth mentioning that a significant part of studies (44.2%, n = 8)^(29,34,36,39,40,44,48,50)^ did not address the adverse effects of the intervention. However, 16.6% (n = 3)^(27,30,51)^ informed that these did not happen, and 38.8% (n = 7)^(32,35,41–43,46,47)^ mentioned having observed adverse effects, being attributed to some discomfort with the acupressure bracelets^(41,43,47)^. Tiredness^(46)^, headache^(42,46)^, anxiety^(42)^, sleep disturbances^(46)^, weight on arms^(46)^, altered taste^(46)^, bruising^(41,46)^, pressure on nose^(46)^, and irritation^(32,35)^have also been reported. Despite this, the majority of studies^(41–43,46,47)^ did not suffer from participant dropouts related to perceived adverse effects.

**Figure 2:**
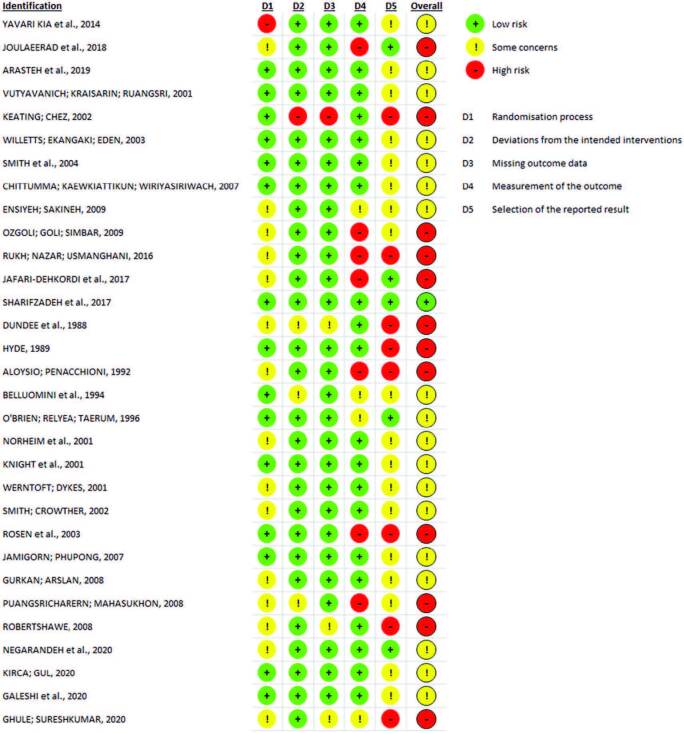
Risk of bias assessment, divided by domains, of the articles included in the systematic review.

Figure 2 shows risk of bias assessment of studies included in the final sample. Most studies (n = 18; 58.1%) had some concerns, while 38.7% were classified as high risk of bias, and 3.2% (n = 1), low risk of bias.

**Figure 3:**
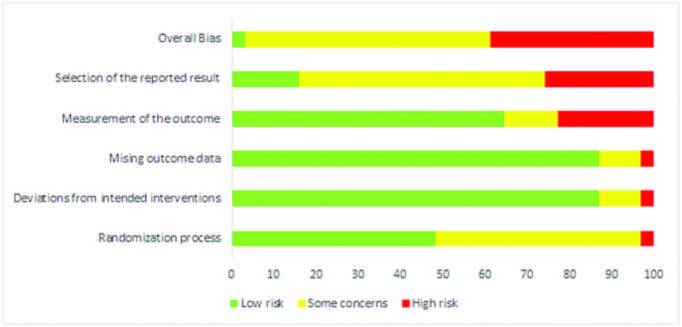
Risk of bias assessment of articles included in the systematic review.

Article analysis, using the tool, allowed the assessment and visualization of risk of bias and, partially, of the methodological quality. As suggested by Cochrane, the risk of bias opinion of the clinical trials included in the systematic review is synthetized in Figure 3.

## DISCUSSION

The results obtained through the systematic review, through the individual analysis of included studies, reflect that the most used ICPH for gestational nausea and vomiting management are aromatherapy, performed with lemon essential oil, phytotherapy, using capsules of ginger and acupuncture, with CS6 point acupressure.

Regarding article inclusion criteria, participants’ age between 16 and 44 years old is possibly related to the consensus established for fertile/reproductive age, between 15 and 49 years old^(52)^. The GA of six to 12 weeks corroborates the findings that gestational nausea and vomiting usually occur between the sixth and twelfth week of gestation^(2)^.

Concerning assessment instrument use, there was great variability in the measurement of outcomes, due to the fact that there is no established COS for studies of nausea and vomiting. The COS seeks to minimally standardize which results should be measured and reported in a given area of research, being associated with the instruments that will be used to assess the outcomes^(53)^. It is noted that this heterogeneity can result in repeated measures of outcome and inconsistency in instrument quality in terms of reliability and validity. Thus, there is an impasse in comparing the results, which makes systematic review studies and meta–analyses difficult. Consensus regarding the instruments and results for a given thematic area of research has relevance to improve the quality of clinical trials, directly impacting evidence–based practice^(54)^.

In studies that applied aromatherapy, there was a tendency to use almond oil as a carrier in the preparation of essential oil^(23,24)^, recommending that two to five drops of this solution be dripped on cotton^(23,24)^, and breathed three times^(23–25)^, repeating every five minutes, if necessary^(23–25)^, for four days, according to the perceived need^(23–25)^. Previous evidence was found in the literature for some of these established topics. The National Association for Holistic Aromatherapy recommends that, for direct inhalation, one should use three to five drops of the oil in question and inhale about twice, suggesting three slow and deep inhalations^(55)^. It is noteworthy that aromatherapy obtained 67% (n = 2) of results superior to the control, achieved with lemon essential oil use. When compared with the current literature, it is noted that the effectiveness of the antiemetic action of this compound had already been observed through aromatherapy^(13)^. Other studies have investigated this ICPH for nausea and vomiting control, however, in the postoperative context, they concluded that it may be effective for this purpose^(56)^.

Regarding phytotherapy, in most studies, ginger capsules were offered, with dosage around one gram per day^(6,13,26,28,33,38,45)^ for four days^(6,13,26,28,31,37)^. In a meta–analysis that included 508 participants, divided into six studies of satisfactory methodological quality, it was observed that one gram of ginger per day for at least four days resulted in a five–fold improvement in gestational nausea and vomiting scores^(57)^. Another meta-analysis, involving 1,278 pregnant women with nausea and vomiting and hyperemesis gravidarum, found that dosing up to one and a half grams daily for four days was a safe and effective intervention for gestational nausea^(58)^. It should be noted that interventions with this ICPH obtained 90% (n = 9)^(6,13,26–28,31,37,38,45)^ of results superior to the control, and 88.8% of these (n = 8)^(6,13,26,28,31,37,38,45)^ used some form of ginger. Research indicates the efficacy of ginger to control nausea and vomiting during pregnancy, especially in the case of mild symptoms, and also discusses minimal adverse effects^(13,59)^. A meta–analysis conducted on the subject concluded that ginger is an effective treatment for this purpose^(57)^.

Regarding the effectiveness of quince syrup, a study included in this review^(27)^ was the first to study this fruit for managing nausea and vomiting so that more research is needed, in order to deepen the knowledge about this herbal medicine. Informally, this is used for gastrointestinal disorders, evidencing in the literature the anti–reflux, antinauseant and antiemetic properties^(60)^. More recently, quince proved to be as effective as ranitidine for treating gastroesophageal reflux disease in pregnancy^(61)^.

Acupuncture was the most heterogeneous in relation to the intervention protocol. It is noted that acupressure, through bracelets at CS6 point, of continuous use, for four days, was the most frequent intervention configuration. However, no previous studies of relevance were found to support this treatment protocol. This finding is possibly related to the fact that the standardization of treatments is a critical node for Traditional Chinese Medicine techniques, since it is contrary to the principle of individuality, which values planned therapy, according to each individual’s characteristics^(62)^.

Acupuncture techniques at CS6 point are widely reported in the literature for nausea and vomiting in various audiences^(63,64)^. Another meta–analysis that sought to demonstrate the effectiveness of various acupuncture techniques at this point for preventing postoperative nausea and vomiting in children concluded that this intervention reduces the incidence of these symptoms as well as use of antiemetics^(63)^. Another meta–analysis showed that acupressure and acupuncture were associated with better control of pregnancy hyperemesis symptoms than standard drug treatment^(64)^.

Regarding CS6 acupressure, a study that synthetized the Cochrane conclusions showed that this intervention proved to be as effective as antiemetic drugs, but with fewer adverse and more transient effects in the postoperative period^(65)^. Another study concluded that CS6 and R21 acupressure may be recommended for this purpose, ensuring efficacy and safety^(13)^.

Low–level nerve stimulation at this same point (CS6) was recommended by meta–analysis, developed to prevent postoperative nausea and vomiting in breast surgeries^(66)^. An RCT conducted with women undergoing cesarean section under combined spinal–epidural anesthesia found that CS6 point stimulation was as effective as routine prophylactic intravenous antiemetic treatment^(67)^.

Regarding the validity assessment of the results of the studies involved in this review, it is noteworthy that CONSORT^(21)^ and the Cochrane tool for assessing risk of bias were used. Through these, we sought to reflect on risk of bias, noting that most studies (n = 18; 58.1%) fit into the category “some concern”. Thus, it is understood that there are possible flaws in the synthetized evidence, which contributes to the uncertainty of the overall evidence^(22)^. It should be noted that this result is related to the fact that the overall risk of bias corresponds to the lowest judgment in any of the domains and not to the result obtained in most domains.

Given the above, it is observed that more well–designed scientific evidence is still needed on this topic. These should seek to improve the quality of reports, preferably those using recommended instruments, such as CONSORT^(21)^. Another benefit of clear design and report is the reflection in the reduction of risk of bias in studies of later reviews, favoring the certainty of the evidence found. Moreover, it is vital that a COS for studies of gestational nausea and vomiting be constructed in order to standardize the outcomes measured and the instruments used, minimizing the heterogeneity of the variables assessed, enabling more cohesive review studies and, consequently, the performance of meta–analyses.

There are also some study limitations, such as failure to conduct the meta–analysis and, consequently, to assess evidence quality, due to the heterogeneity of included studies in relation to: method, sample design, outcome assessment method and statistical analysis, even among those dealing with the same ICPH; non–inclusion of gray literature in the sources of information, due to the number of articles identified at first; and uncertainty of the conclusions obtained, due to risk of bias of included studies. However, this review presents data from individual articles with clinical significance for the management of pregnant women with symptoms of nausea and vomiting, with useful findings for health professionals who provide care to this clientele.

## CONCLUSION

The synthesized and analyzed evidence points to three categories of ICPH used for the control of gestational nausea and vomiting, aromatherapy, phytotherapy and acupuncture. Specifically, aromatherapy with lemon essential oil (drip two to five drops and breathe three times, repeat every five minutes, if necessary, for four days and as needed), ginger capsules (one gram a day, for four days) and CS6 acupressure (through bracelets, continuously for four days) were the interventions that proved most effective in the individual assessment of studies. However, it was not possible to perform a meta–analysis to affirm the overall effect of each of these interventions on the outcome assessed.

Adverse effects were reported in 45.2% of studies. Most symptoms were mild and transient, and no statistical difference was observed between the intervention and control groups so that these did not result in withdrawal of participants.

The results of this review can support reflections by health professionals and, especially, obstetric nurses, on the use of these ICPH in the treatment of pregnant women with nausea and vomiting, with a view to contributing to evidence–based use and demystification of these practices, promoting, above all, a better quality of life for this very specific population.
